# Supplementary material for: Tempering of cocoa butter and chocolate using minor lipidic components
Source: Nat Commun. 2021 Aug 31;12:5018. doi: 10.1038/s41467-021-25206-1 (PMC8408162; doi:10.1038/s41467-021-25206-1)
Supplement: Supplementary file 1 — Supplementary Information [file 41467_2021_25206_MOESM1_ESM.pdf]

Tempering of cocoa butter and chocolate using minor lipidic components

Jay Chen, Saeed M. Ghazani, Jarvis A. Stobbs, & Alejandro G. Marangoni

Supplementary Information

Supplementary Table 1: Fatty acid profile of unrefined cocoa butter and monoacylglycerol additives determined by gas-liquid chromatography.

Cocoa Butter		GMO		GMP		GMS	
Fatty Acid	Area %	Fatty Acid	Area %	Fatty Acid	Area %	Fatty Acid	Area %
C16:0	25.40	C16:0	6.45	C14:0	1.24	C16:0	11.10
C18:0	37.30	C18:0	1.81	C16:0	56.94	C18:0	88.33
C18:1	33.54	C18:1	81.8	C18:0	40.52	C20:0	0.57
C18:2	2.51	C18:2	7.1	C20:0	0.43		
C20:0	1.24						

Supplementary Table 2: Phosphorus concentrations in cocoa butter before and after neutralization and bleaching as determined by AOCS Official Method Ca 12-55. Unrefined average represents the mean of 3 replicates.

Before Degumming:	Unrefined Average	Neutralized	Bleached
**Mass of CB (g)**	3.13	3.08	3.12
**Absorbance Trial 1**	0.1737	0.0053	0.0089
**Absorbance Trial 2**	0.1734	0.0059	0.0098
**Absorbance Trial 3**	0.1740	0.0057	0.0102
**Average Absorbance**	0.1737	0.0056	0.0096
**Phosphorus Content (mg)**	0.0283	-0.0003	0.0004
**Phosphorus %**	0.0088	-0.0004	-0.0001
**Equivalent Phosphatides %**	0.2629	-0.0109	-0.0042

Supplementary Table 3: Free fatty acid concentrations in cocoa butter before and after neutralization and bleaching as determined by AOCS Official Method Ca 5a-40. Unrefined average represents the mean of 3 replicates.

Trial	Unrefined Average	Neutralized	Bleached
**CB Mass (g)**	28.20	28.39	29.70
**NaOH (mL)**	3.23	0.21	0.12
**% as Oleic acid**	1.646	0.106	0.058
**% as Lauric acid**	1.168	0.075	0.041
**% as Palmitic acid**	1.495	0.096	0.053

Supplementary Table 4: Characteristic wide-angle powder X-ray diffraction short spacings for cocoa butter in crystal polymorphic forms of IV, V and IV.

Polymorph	Short-spacing (Å)*				
IV	4.35 (s)	4.15 (s)	3.97 (m)	3.81 (m)	
V	4.58 (vs)	3.98 (m)	3.87 (m)	3.75 (m)	3.67 (m)
VI	4.61 (vs)	4.06 (m)	3.96 (m)	3.88 (m)	3.71 (s)

*vs, s, m, w represents "very strong", "strong", "medium" and "weak" peak intensities.

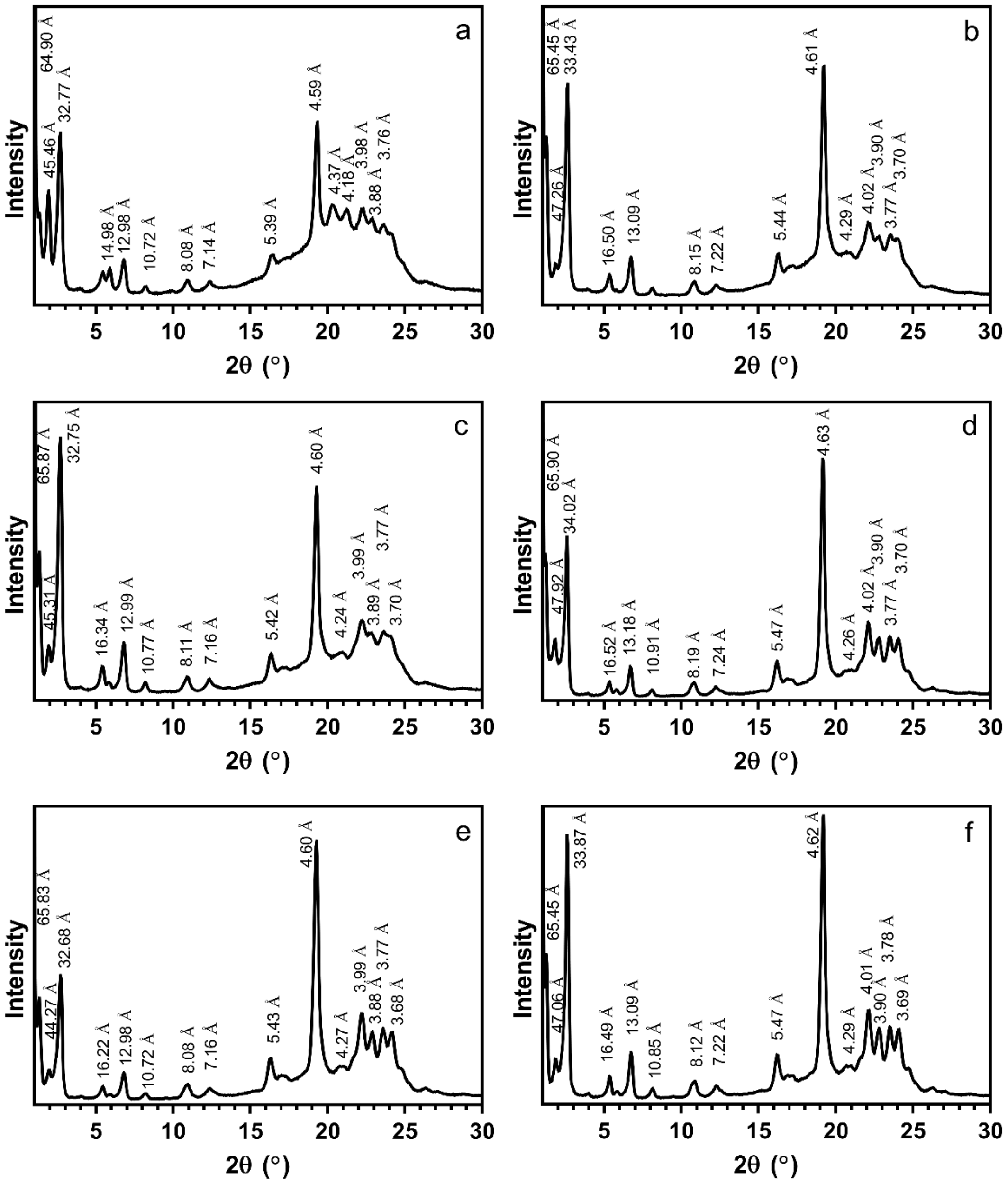

Supplementary Figure 1: Powder X-ray diffraction patterns for refined cocoa butter with added minor lipidic components. Samples included refined CB with added 0.5% (w/w) of (a) glycerol monostearate, (b) glycerol monopalmitate, (c) glycerol monooleate, (d) stearic acid, (e) palmitic acid, and (f) oleic acid. Samples were crystallized statically at 23°C for 24 hours

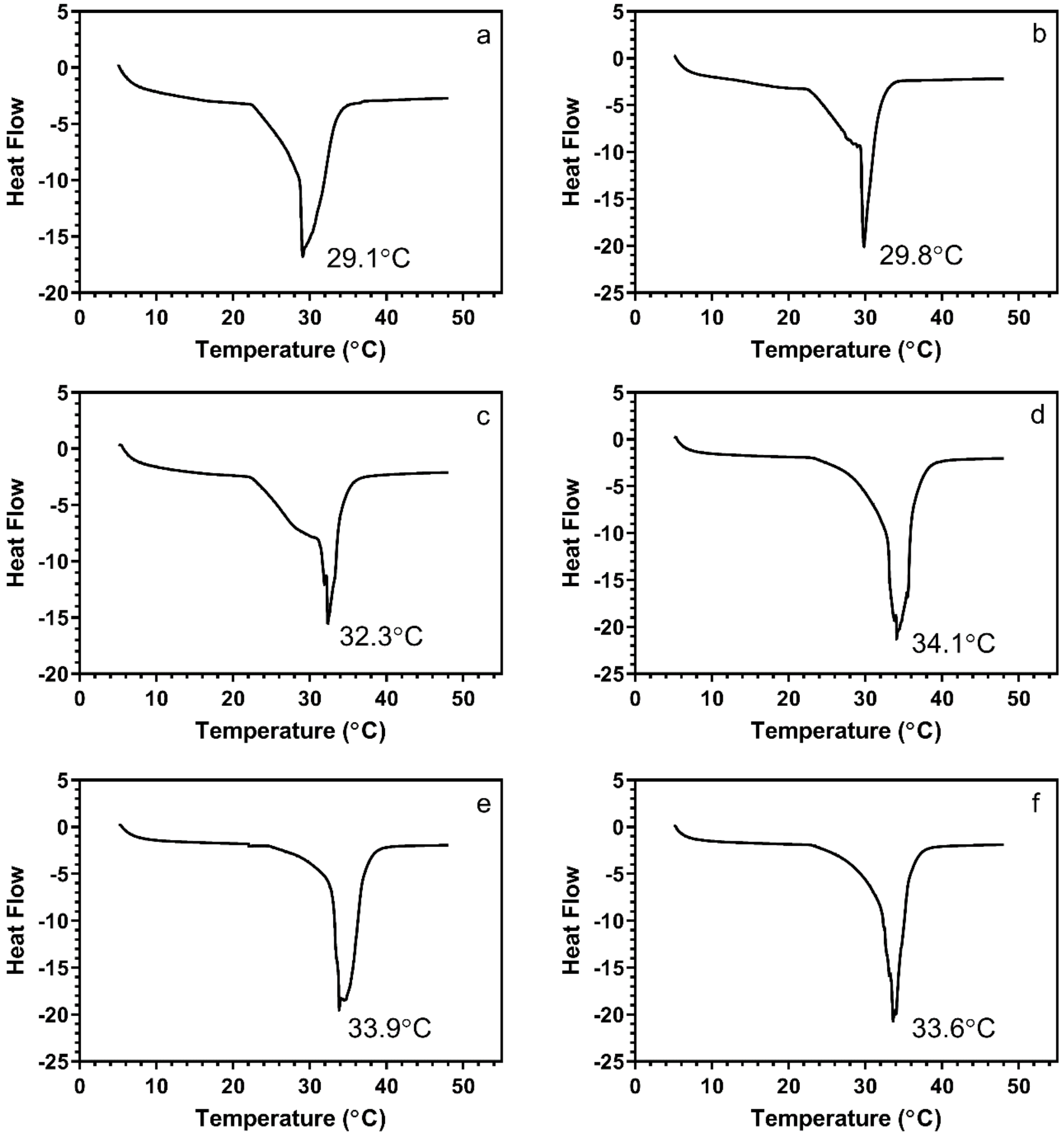

Supplementary Figure 2: Differential scanning calorimetry melting thermograms and peak melting temperatures for refined cocoa butter with added minor lipidic components. Samples included refined CB with added 0.5% (w/w) of (a) glycerol monostearate and (b) glycerol monopalmitate, (c) glycerol monooleate, (d) stearic acid, (e) palmitic acid and (f) oleic acid. Samples were crystallized statically at 23°C for 24 hours and scanned using a heating rate of 5°C/min

Supplementary Table 5: Avrami constants and crystallization half-times calculated by nonlinear regression ($SFC = SFC_{max}(1 - e^{-k_A t^n})$) of samples crystallized statically at 23°C (n=3, ± standard error). Values with the same superscript letter within a row are not statistically different (P>0.05).

Sample	Unrefined	Refined	DMPC	DPPE
k_A (min^{-n})	1.91±0.43E-09^{a}	5.58±1.85E-07^{a}	3.57±1.79E-06^{b}	3.52±1.90E-11^{a}
n	3.51 ± 0.06^{a,b}	2.67 ± 0.06^{a}	3.23 ± 0.71^{a,b}	4.88 ± 0.17^{c}
SFC_{max} (%)	49.1 ± 0.2^{a}	59.5 ± 0.2^{b}	56.9 ± 0.8^{c}	55.4 ± 0.3^{c,d}
Half-time (min)	281.9 ± 6.0^{a}	200.6 ± 3.7^{b,c}	112.3 ± 6.5^{d}	141.7 ± 3.2^{d,e}

Supplementary Table 5 continued

Sample	GMS	GMP	GMO	Stearic	Palmitic	Oleic
k_A (min^{-n})	2.15±1.33E-10^{a}	2.65±1.22E-09^{a}	1.70±1.50E-08^{a}	3.34±2.65E-09^{a}	4.36±2.66E-08^{a}	4.39±0.77E-08^{a}
n	4.23 ± 0.25^{b,c,d}	3.64 ± 0.15^{a,c}	3.66 ± 0.22^{a,c}	3.92 ± 0.18^{a,c}	3.33 ± 0.16^{a,d}	3.19 ± 0.04^{a,d}
Y_{max} (%)	55.9 ± 0.3^{c,d}	55.0 ± 0.4^{c,d}	54.6 ± 0.3^{c,d}	54.7 ± 0.3^{c,d}	54.2 ± 0.3^{d}	56.3 ± 0.5^{c,d}
Half-time (min)	217.6 ± 8.2^{b}	226.5 ± 7.8^{b}	164.8 ± 4.3^{e,f}	163.1 ± 3.4^{e,f}	170.5 ± 4.6^{e,f}	183.8 ± 1.8^{c,f}

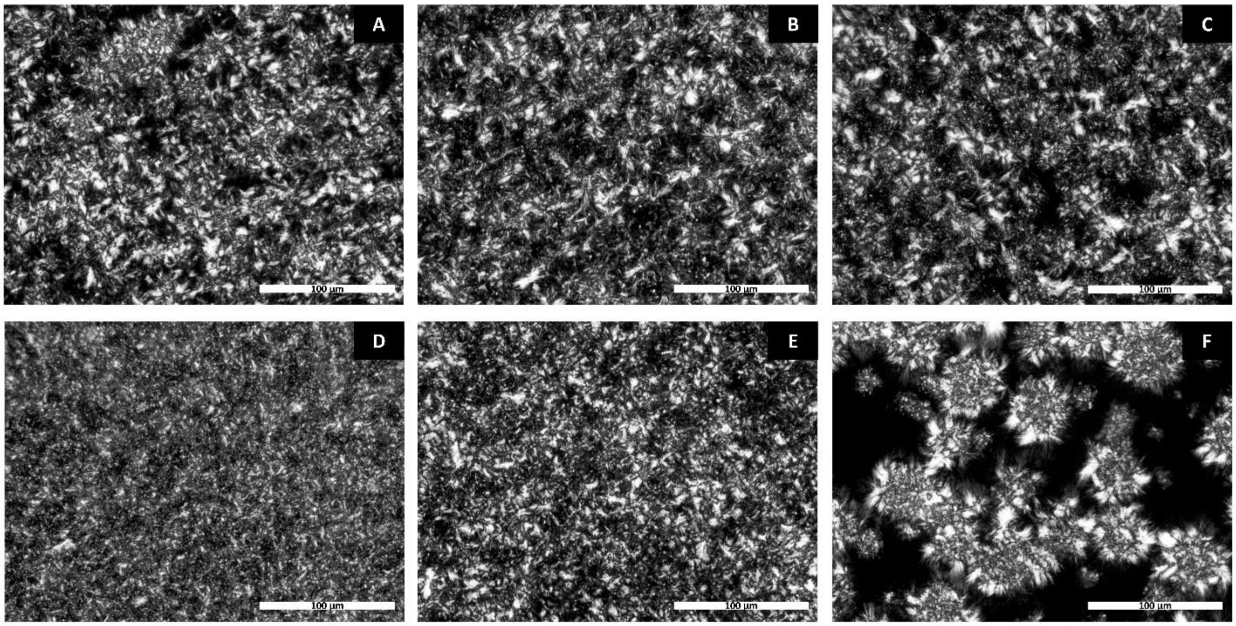

Supplementary Figure 3: Polarized light microscopy images of refined cocoa butter with added minor lipidic components. Samples included refined CB with added 0.5% (w/w) of (a) glycerol monostearate and (b) glycerol monopalmitate, (c) glycerol monooleate, (d) stearic acid, (e) palmitic acid and (f) oleic acid. Samples were crystallized statically at 23°C for 24 hours. The horizontal magnification bar at the bottom of the images corresponds to 100µm.

Supplementary Table 6: Box-counting fractal dimension of thresholded 8-bit grayscale polarized light micrographs for all samples used in this study. The intensity and contrast of the grayscale images were auto-levelled and thresholding was carried out automatically using a grayscale level of 128.

Trial	1	2	3	4	5	6	Average	SE
**Unrefined**	1.581	1.591	1.630	1.702	1.644	1.628	1.629	0.018
**Bleached**	1.824	1.787	1.778	1.694	1.715	1.732	1.755	0.020
**DMPC**	1.708	1.725	1.725	1.710	1.716	1.752	1.723	0.007
**DPPE**	1.809	1.799	1.710	1.674	1.627	1.689	1.718	0.029
**GMS**	1.719	1.740	1.620	1.609	1.736	1.689	1.685	0.024
**GMP**	1.694	1.648	1.704	1.694	1.645	1.622	1.668	0.014
**GMO**	1.785	1.691	1.627	1.598	1.630	1.624	1.659	0.028
**Stearic**	1.615	1.603	1.650	1.729	1.584	1.555	1.623	0.025
**Palmitic**	1.568	1.612	1.685	1.782	1.782	1.729	1.693	0.036
**Oleic**	1.567	1.434	1.695	1.743	1.704	1.695	1.639	0.048

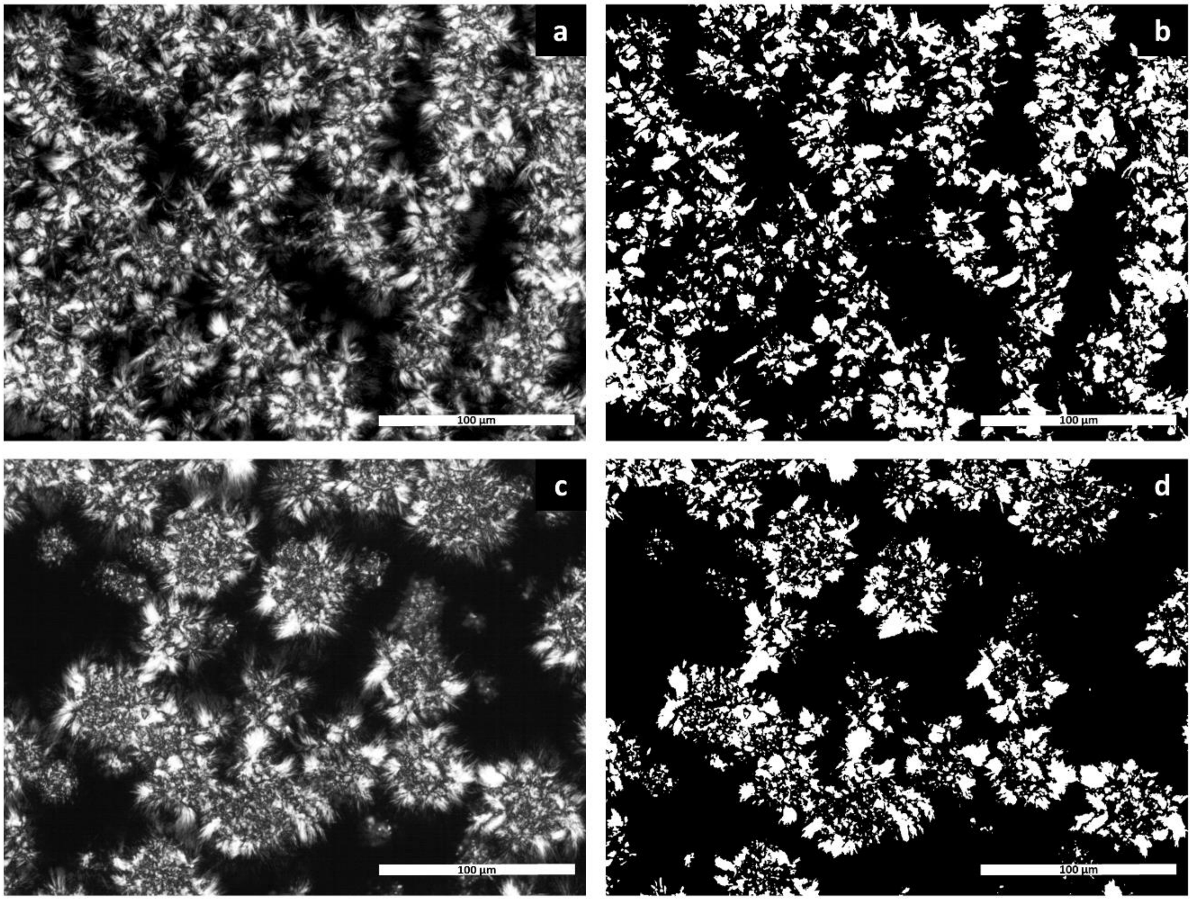

Supplementary Figure 4: Polarized light microscopy images of refined cocoa butter with added minor lipidic components. The 8-bit grayscale micrographs with 256 shades of gray were automatically thresholded using a grayscale level of 128. Here we show the conversion of the grayscale image of (a) refined cocoa butter into its thresholded version (b), as well as the conversion of the grayscale image of (c) refined cocoa butter with added 0.5% oleic acid to its thresholded counterpart (d). Before thresholding, intensity levels and contrast were auto-levelled. These thresholded images were then used for the box-counting analysis. The horizontal magnification bar at the bottom of the images corresponds to 100µm.
