# Supplementary material for: Tempering of cocoa butter and chocolate using minor lipidic components
Source: Nat Commun. 2021 Aug 31;12:5018. doi: 10.1038/s41467-021-25206-1 (PMC8408162; doi:10.1038/s41467-021-25206-1)
Supplement: Supplementary file 3 — Description of Additional Supplementary Files [file 41467_2021_25206_MOESM3_ESM.pdf]

Description of Additional Supplementary Files

File Name: Supplementary Movie 1
Description: Volume rendering camera orbit of synchrotron micro-CT data for a molten and recrystallized commercial chocolate sample with added DMPC (DMPC)

File Name: Supplementary Movie 2
Description: Volume rendering camera orbit of synchrotron micro-CT data for a molten and recrystallized commercial chocolate sample with added DPPE (DPPE)

File Name: Supplementary Movie 3
Description: Volume rendering camera orbit of synchrotron micro-CT data for a fresh commercial chocolate sample (Lindt-F)

File Name: Supplementary Movie 4
Description: Volume rendering camera orbit of synchrotron micro-CT data for a molten and recrystallized commercial chocolate sample (Lindt-R)
